# Supplementary figures and images for: Vocal complexity in the long calls of Bornean orangutans
Source: PeerJ. 2024 May 14;12:e17320. doi: 10.7717/peerj.17320 (PMC11100477; doi:10.7717/peerj.17320)

**Figure S1.** Example of annotated spectrogram

**
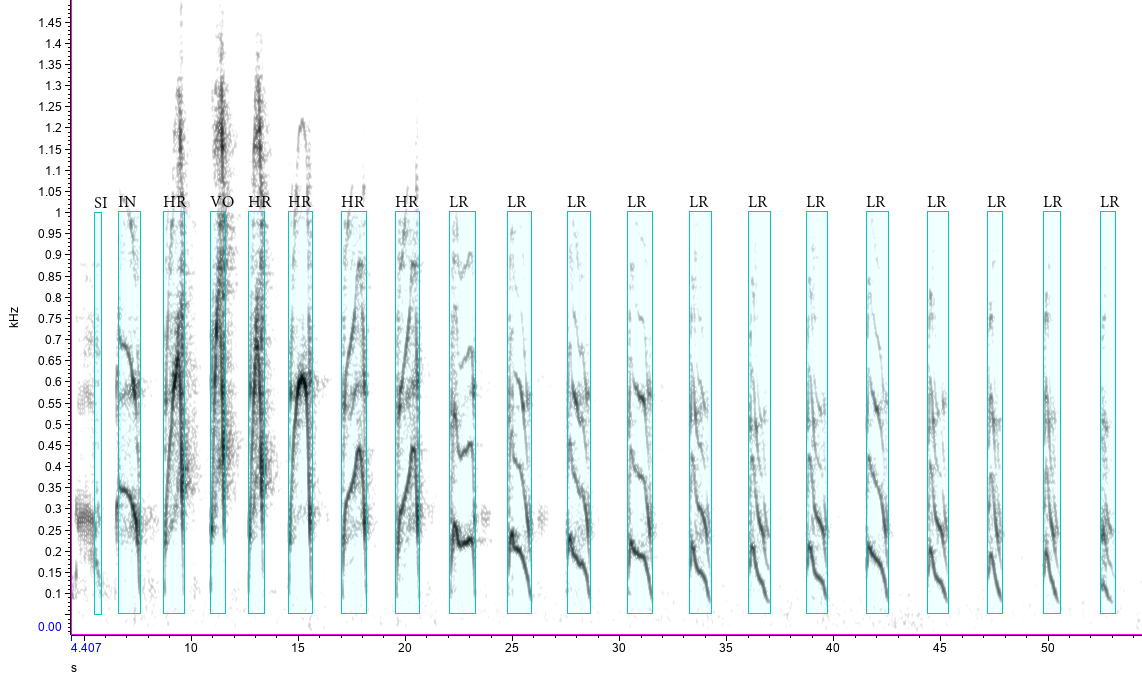
**

Supplement: Supplemental Information 2 [file peerj-12-17320-s002.docx]
