# Supplementary material for: Vocal complexity in the long calls of Bornean orangutans
Source: PeerJ. 2024 May 14;12:e17320. doi: 10.7717/peerj.17320 (PMC11100477; doi:10.7717/peerj.17320)

**Figure S2.** Boxplots of features that differed across human-labeled pulses (upper left), affinity propagation clusters (upper right), and typical calls in fuzzy clusters (lower left) for each of the following influential features: a) center frequency, b) peak frequency, c) mean peak frequency, d) third quartile frequency, e) first quartile frequency.


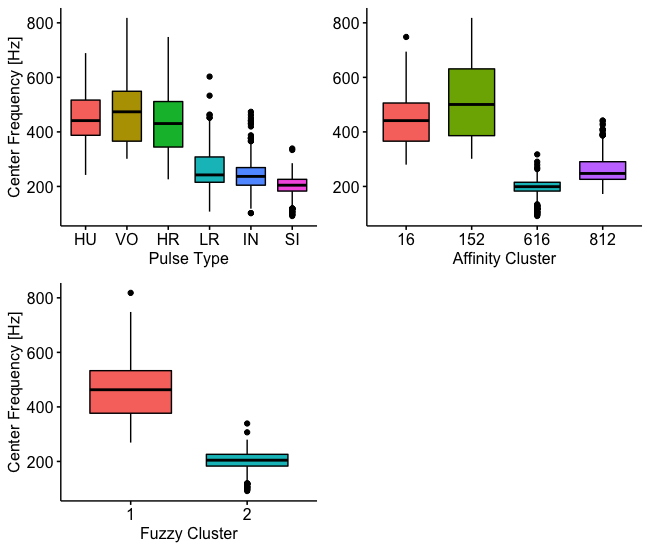


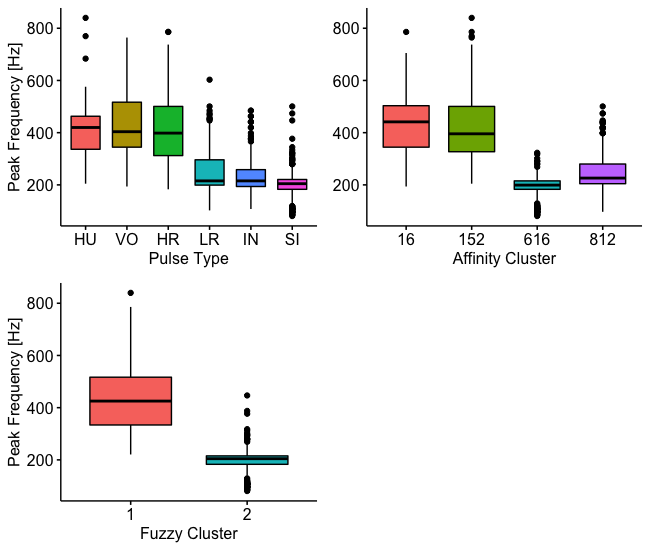


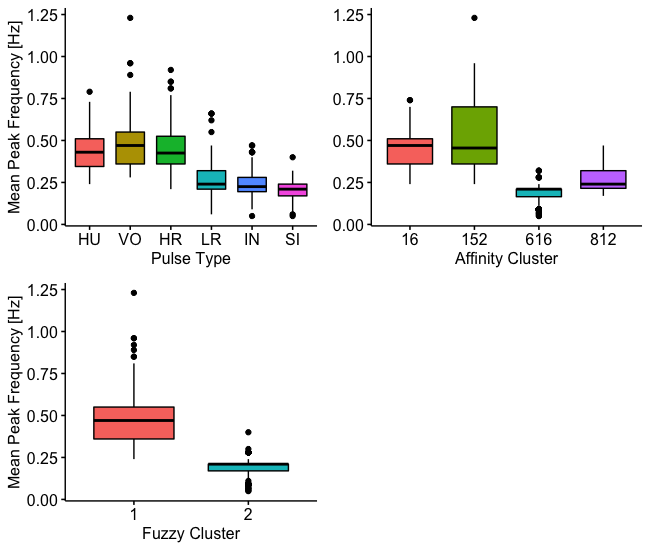


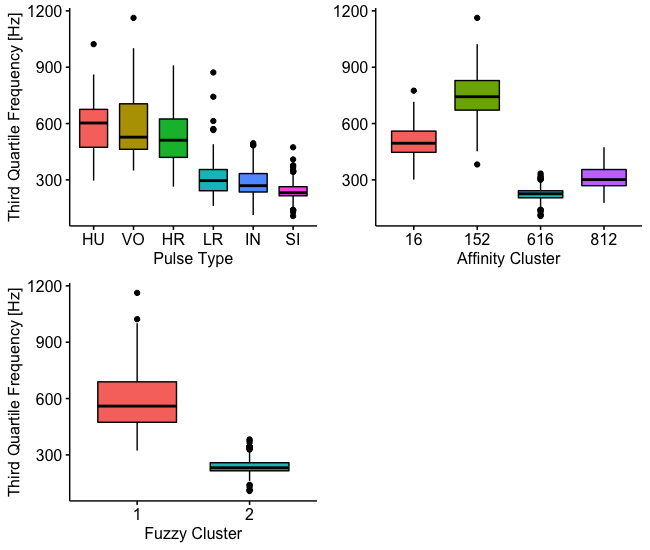


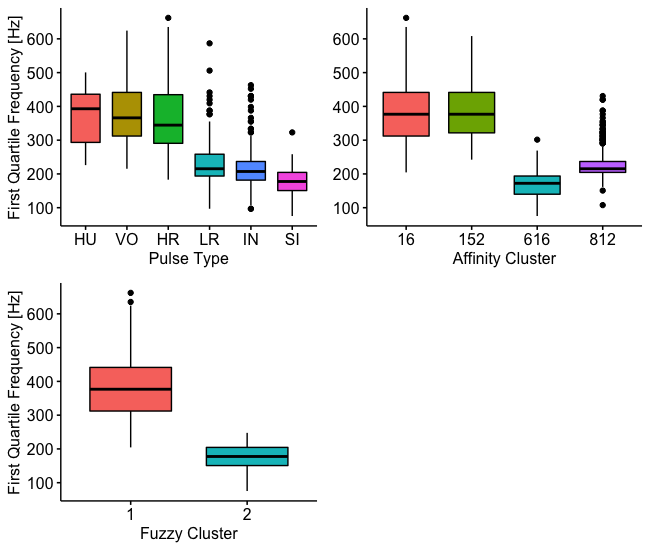

Supplement: Supplemental Information 3 [file peerj-12-17320-s003.docx]
