# Supplementary material for: Vocal complexity in the long calls of Bornean orangutans
Source: PeerJ. 2024 May 14;12:e17320. doi: 10.7717/peerj.17320 (PMC11100477; doi:10.7717/peerj.17320)

**Figure S4.** Histograms showing bootstrapping results across 25 iterations within 2–40 randomly sampled features showing: a) distribution of the number of clusters identified by affinity propagation and b) distribution of the number of clusters identified by fuzzy clustering.


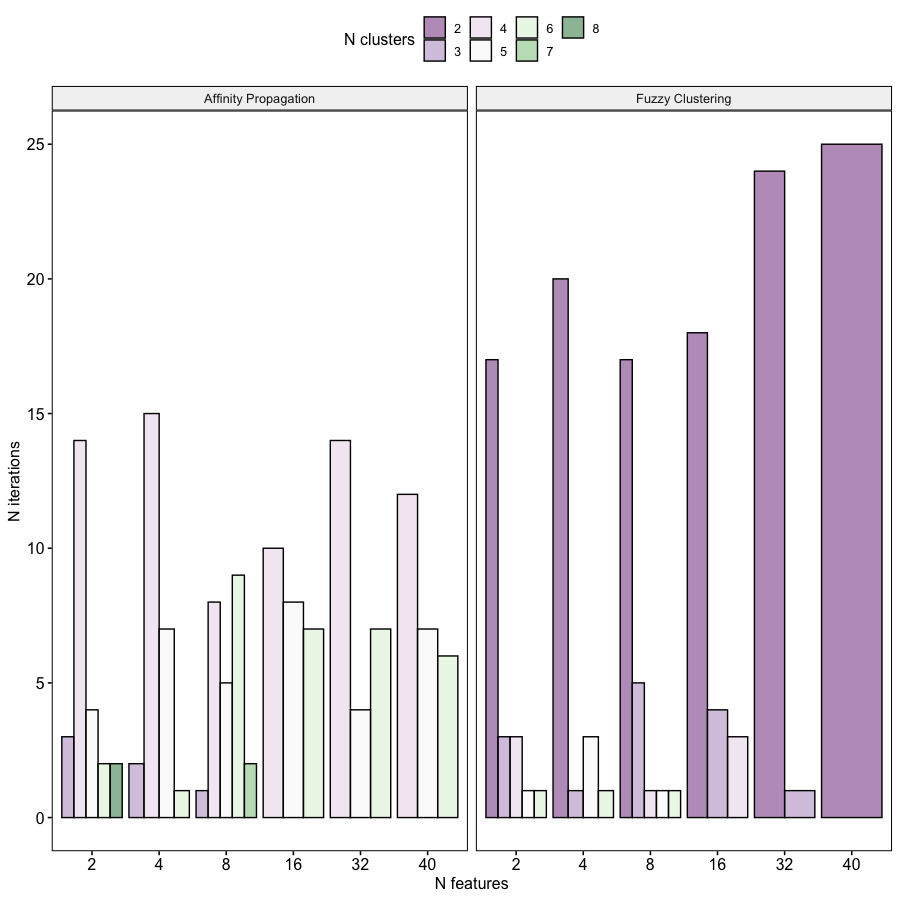

Supplement: Supplemental Information 5 [file peerj-12-17320-s005.docx]
